# Microwave ablation for high-risk pulmonary nodules in patients infected with the Omicron variant of Sars-Cov-2 within 3 months: a retrospective analysis of safety and efficacy

**DOI:** 10.3389/fonc.2024.1445245

**Published:** 2024-10-07

**Authors:** Yuxian Chen, Yang Li, Hong Meng, Chunhai Li, Fanlei Kong

**Affiliations:** ^1^ Department of Radiology, Qilu Hospital of Shandong University, Jinan, China; ^2^ Department of Medical Oncology, The People’s Hospital of Zouping City, Binzhou, China

**Keywords:** microwave ablation, high-risk pulmonary nodules, Omicron variant, safety, efficacy

## Abstract

**Introduction:**

To evaluate the safety and efficacy of microwave ablation (MWA) for high-risk pulmonary nodules in patients infected with the Omicron variant within 3 months, a retrospective study was conducted.

**Methods:**

The study included patients with multiple high-risk nodules who underwent CT-guided MWA from April 2022 to April 2023. Patients were divided into an observation group and a control group. The primary endpoints were postoperative complications and hospital length of stay, while the secondary endpoint was progression-free survival (PFS).

**Results:**

A total of 157 patients were included in the analysis, with 64 in the observation group and 93 in the control group. No deaths occurred within 30 days after MWA. In the observation group, the median follow-up time was 7 months, during which 5 patients experienced disease progression after MWA, including 3 cases of pulmonary metastases. Complications were primarily pneumothorax, pleural effusion, and hemorrhage, with an incidence rate of 57.8%, which was statistically significant (p=0.005). The median length of hospital stay was 5 days for the observation group and 6 days for the control group. There was no statistically significant difference in PFS between the two groups after the removal of lung metastases (p=0.265).

**Discussion:**

CT-guided MWA is an alternative treatment for patients with high-risk lung nodules who have been infected with Omicron within the past 3 months.

## 1 Introduction

Since the outbreak of coronavirus disease 2019 (COVID-19), the number of canceled and postponed surgeries has exceeded ten million cases, and corresponding clinical recommendations and consensus have been continuously introduced internationally (1). As severe acute respiratory syndrome coronavirus 2 (SARS-CoV-2) continues to evolve and mutate, the Omicron variant has become the predominant strain worldwide (2, 3). Due to the highly infectious nature of Omicron strains, many patients with high-risk lung nodules are also experiencing significant infections. SARS-CoV-2 infection can lead to multi-system disease, including chronic pulmonary dysfunction, myocardial inflammation, renal impairment, psychological distress, and chronic fatigue, potentially resulting in both short- and long-term sequelae (4–6). These complications can impact postoperative recovery and must be considered when developing a safe surgical program. Additionally, patients with high-risk lung nodules require prompt management to prevent disease progression and reduce anxiety. Microwave ablation (MWA) is a novel, minimally invasive technique that offers potentially effective solutions for primary and metastatic lung tumors, providing excellent local control and improving patient survival prognosis (7–9). However, there is relatively little experience regarding the optimal timing of MWA following an Omicron infection, and there is a lack of reports on the impact of MWA on the clinical prognosis of patients. Therefore, this study aimed to evaluate the safety and efficacy of MWA in patients with high-risk pulmonary nodules within three months of Omicron infection and to provide a theoretical basis for further standardization of the treatment process.

## 2 Materials and methods

### 2.1 Patients

At the end of 2022, a significant number of patients were infected following the adjustment of our COVID-19 control policy. This study included a total of 157 patients with high-risk lung nodules who were treated with MWA at our hospital between August 2022 and August 2023. Among these patients, 64 individuals with positive nucleic acid tests within 3 months prior to MWA treatment were assigned to the observation group, while the remaining 93 patients constituted the control group. High-risk lung nodules were defined as those with pathologically confirmed malignancy or imaging suspicion of malignancy, accompanied by an increase in nodule diameter or solid portion diameter of more than 2 mm, or the presence of a new solid component in the nodule upon follow-up. The observation group comprised 56 primary lesions and 8 lung metastases. Of the primary lesions, 37 were pathology-free; 14 were invasive adenocarcinomas, with 7 post-pulmonary and 3 post-targeted therapy cases; 1 was a squamous carcinoma, post-pulmonary; 4 were adenocarcinomas *in situ*, with 2 post-pulmonary, 1 post-particle implantation, and 1 post-laryngeal case; and the lung metastatic tumors included 2 metastases of renal carcinoma, 2 metastases of sigmoid colon carcinoma, 1 metastasis of endometrial carcinoma, and 3 metastases of rectal carcinoma, all of which were controlled at the primary site of origin. Detailed patient information is provided in **Table 1**. This retrospective study was approved by our institutional review board, and the requirement for informed consent was waived. Furthermore, written informed consent was obtained from all patients prior to MWA.

**Table 1:** Clinical characteristics of patients in two groups.

Variables	Observation group	Control group
Patients, n (%)	64	93
Sex, n (%)		
Male	24 (37.5%)	44 (47.3%)
Female	40 (62.5%)	49 (52.7%)
Age (y), average (range)	60.3 (34-88)	61.4 (29-82)
Smoking history, n (%)		
Yes	18 (28.1%)	30 (32.3%)
No	46 (71.9%)	63 (67.7%)
Location, n (%)		
Upper left	19 (29.2%)	35 (37.6%)
Lower left	7 (10.8%)	6 (6.5%)
Upper right	24 (36.9%)	28 (30.1%)
Middle right	0	9 (9.7%)
Lower right	15 (23.1%)	15 (16.1%)
Lesion number, n (%)		
1	63 (98.4%)	92 (98.9%)
2	1 (1.6%)	1 (1.1%)
Size, n (%)		
<1cm	34 (52.3%)	43 (45.7%)
1-3cm	31 (47.7%)	50 (53.2%)
>3cm	0	1 (1.1%)
Stage, n (%)		
I	57 (87.7%)	87 (93.5%)
II	0	1 (1.1%)
III	0	1 (1.1%)
IV	8 (12.3%)	4 (4.3%)
Pathology		
Adenocarcinoma	14 (21.9%)	35 (37.6%)
Adenocarcinoma in situ	4 (6.3%)	5 (5.4%)
Squamous cell carcinoma	1 (1.5%)	2 (2.2%)
Large cell carcinoma	0	0
Small cell carcinoma	0	1 (1.1%)
Lung metastasis	8 (12.5%)	3 (3.2%)
Non-pathological	37 (57.8%)	47 (50.5%)

### 2.2 Pre-ablation assessment

All patients underwent routine evaluation by an interventional radiologist prior to MWA, which included assessing medical history, conducting a physical examination, and performing laboratory tests and relevant imaging studies such as chest CT, whole-body positron emission tomography/computed tomography (PET/CT), or both. Routine preoperative electrocardiograms and pulmonary function tests were also conducted. Anticoagulant and antiplatelet medications were temporarily discontinued for a period ranging from 1 day to 1 week, depending on the specific medication, and blood pressure and blood glucose levels were managed to ensure they remained within safe ranges.

### 2.3 MWA procedure

The ECO-100C MWA device was utilized (ECO Microwave Electronic Institute, Nanjing, China; registration standard: YZB/country 3388–2011; China: SFDA certified No.20113251473). The microwave transmission frequency was set at 2450 ± 50 MHz, with an output power range of 0-150 W. The microwave antenna had an effective length of 130-180 mm and an outer diameter of 15G-18G. A water-circulating cooling system was employed to lower the antenna surface temperature. Local anesthesia and preemptive analgesia were administered to the patients.

Prior to the procedure, the patient’s position (supine, lateral, prone, etc.) was determined by analyzing the CT images. Body marking lines were posted, and the level and location of the lesion were identified based on the CT scans. The path of entry for the puncture needle was also planned. The procedure was conducted under aseptic conditions and local anesthesia. The ablation needle was gradually inserted into the lesion under CT guidance, and a cable was used to connect the ablation needle to the microwave ablator and the cold circulatory tube. The ablation time and power were determined based on factors such as the size of the lesion, changes in the lesion, and patient tolerance.

### 2.4 Post-ablation management and follow-up

Complications using the unified standardized SIR grading system (10). Any patient who died within 30 days after the procedure was classified as SIR classification F. A major complication was defined as an event that resulted in significant morbidity and disability, which corresponded to SIR classifications C-E. This included any case where a blood transfusion or interventional drainage procedure was necessary. All other complications were considered minor. All patients were hospitalized for MWA and observation. Immediately after the ablation, a CT scan of the chest was performed to assess the completeness of the ablation and to check for any possible complications such as pneumothorax, hemorrhage, pleural effusion, *etc.* If the ablation was deemed complete and there were no urgent complications to manage, the patient was transferred to the ward for observation. During this time, attention was paid to monitoring for postoperative infections and wound pain, and routine monitoring of heart rate, blood pressure, and oxygen saturation was performed. A chest CT scan was performed 24-48 hours after MWA, and patients were discharged from the hospital 2-3 days after the ablation if there were no complications requiring further treatment. Patients were followed up with chest CT at 1, 3, 6, and 12 months after the procedure and every 6 months thereafter.

### 2.5 Validity

High-risk GGN is evaluated based on the presence of malignant margins and internal structural features, as well as clinical progression, which is generally defined as an increase of more than 2 mm in nodal diameter or the diameter of the solid portion of the nodule, or the presence of a new solid component in the nodule, after a minimum of 3 months of follow-up. Pathological confirmation of malignancy is also required. Technical success is defined as an immediate postoperative CT scan of the chest showing complete coverage of the tumor by the ablation zone and adequate ablation margins. Subject to technical success, local tumor progression (LTP) is determined based on the presence of progressively larger lesions in the ablation zone or nodal enhancement within or at the margins of the ablation zone during CT follow-up. Minimal ablation margin (MM) measurements are performed on tumors that achieve technical success. MM is defined as the shortest distance between the ablated lesion and the edge of the ablation zone measured on CT images scanned immediately after MWA. MM>5mm is defined the end point of MWA.

### 2.6 Statistical analysis

Continuous variables are presented as mean and standard deviation (SD) or median and range, while categorical variables are expressed as frequencies and percentages. The t-test is used for analyzing continuous variables, and the Pearson chi-squared test or Fisher exact test is used for categorical variables. PFS is calculated using the Kaplan-Meier method, and the log-rank test is used to compare differences between groups. Cox regression analysis is used to identify significant prognostic factors. A p-value < 0.05 indicates significant differences. All statistical analyses are performed using SPSS (version 27; SPSS, Inc., Chicago, IL, USA).

## 3 Results

### 3.1 Patients

**Figure 1:**
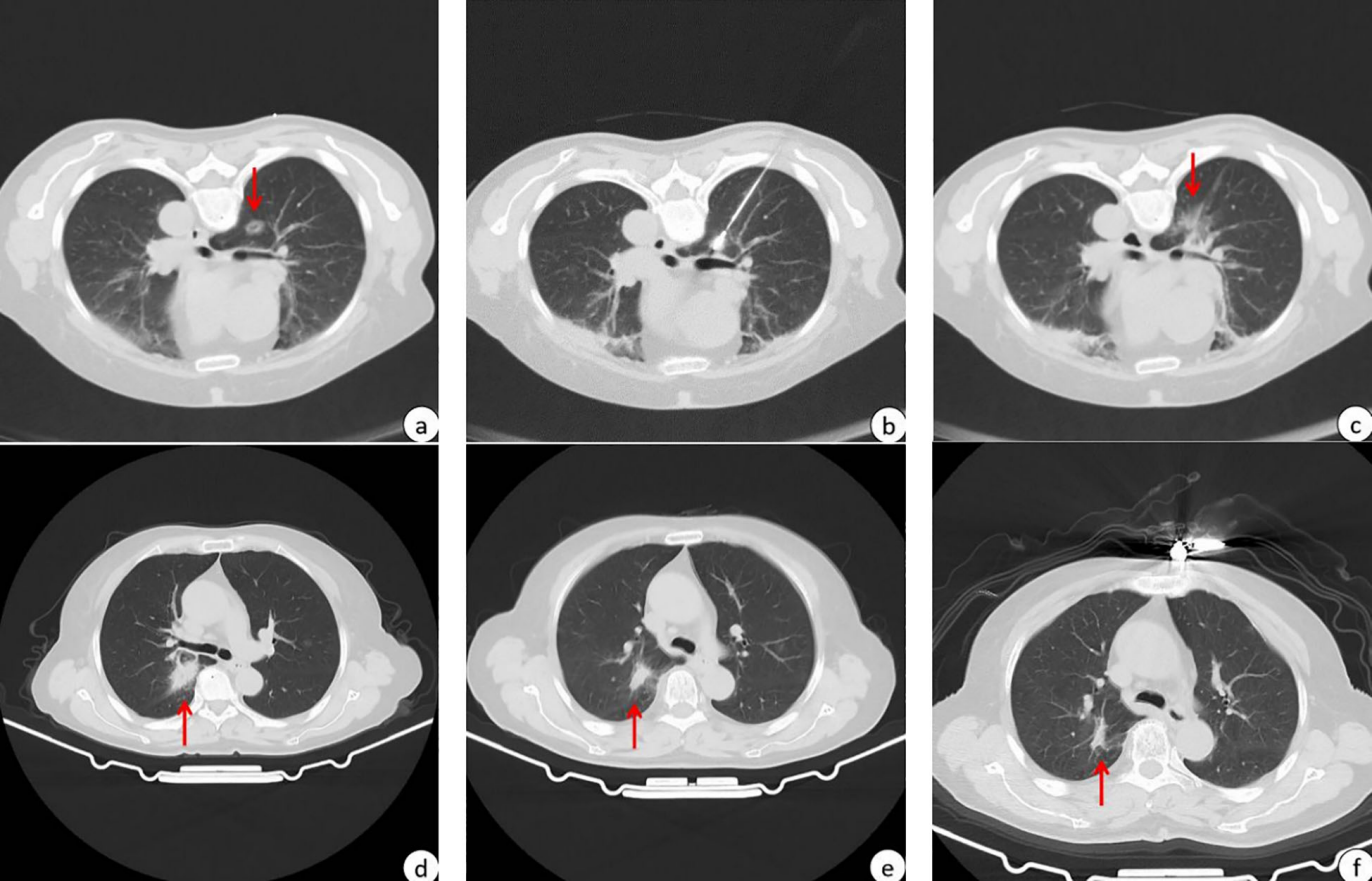
A patient with high-risk pulmonary nodules treated with MWA within 3 months of Omicron infection. The red arrows in the figure point to high-risk pulmonary nodules to be subjected to MWA or post-MWA areas. **(A)** 13.8*10.7 mm GGN in the upper lobe of the right lung before MWA; **(B)** CT scan during MWA; **(C)** after MWA, the halo shadow (36.8*30.2mm) was significantly larger than the lesion; **(D)** imaging performance 1 months after MWA, and post-ablation area 31.2*18.4mm; **(E)** imaging performance 4 months after MWA, and post-ablation area 17.9*10.7 mm; **(F)** imaging performance 10 months after MWA, and post-ablation area 16.7*6.5 mm.

In the retrospective study, 157 patients were treated with MWA, with 64 (40.8%) infected with SARS-CoV-2 and 93 (59.2%) uninfected. All patients in the observation group had preoperative infections, with 17 still showing pneumonia at the time of MWA and 47 having asymptomatic lungs. The average interval from COVID-19 infection to MWA was 59.3 days (an example shown in **Figure 1**). A total of 64 patients in the observation group received 64 MWA treatments for 65 lesions, while 93 patients in the control group received 93 MWA treatments for a total of 94 lesions. All patients received 1 ablative session, with 1 patient in each group receiving 1 MWA for two lesions. The technical success rate was 100%, and MM was measured for all lesions, with 38/64 (59.4%) of tumors having MM > 5 mm and 26/64 (40.6%) having MM ≤ 5 mm (**Table 2**). In the observation group, the last follow-up was on 27 March 2024, and the median follow-up duration in all patients was 7 months (range: 1-13 months), with an average follow-up time of 7.16 months (range: 1-13 months). In the control group, the median follow-up duration was 12 months (range: 1-21 months), with an average follow-up time of 10.6 months (range: 1-21 months). Despite the initial technical success, subsequent CT scans showed progression in the ablation zone in 5 patients in the observation group, including adenocarcinoma *in situ* (n=2) and lung metastases (n=3), with a LTP rate of 7.8% (5/64). All 5 progressive tumors were located in the periphery of the lungs, with 2 of them being adjacent to the pleura (distance from the pleura <10 mm), and 2 of the 5 LTPs appeared in lesions with MM ≤ 5 mm. In the control group, 7 patients had progression in the ablation zone, including adenocarcinoma (n=4), adenocarcinoma *in situ* (n=2), and squamous cell carcinoma (n=1), with an LTP rate of 7.5% (7/93). All of the progressed tumors were located in the periphery of the lungs, with 4 of them being adjacent to the pleura (distance from the pleura <10 mm), and 1 of the 7 LTPs appeared in lesions with MM ≤ 5 mm. The mean size of the tumors in the observation and control groups was 11.8 mm (range 5.5-28.9 mm) and 12.4 ± 5.8 mm (range 5.0-37.0 mm), respectively.

**Table 2:** Result characteristics of patients in two groups.

Characteristic	Observation group	Control group
MM>5mm MM<5mm Follow-up time (m)	38 (59.4%) 26 (40.6%) 7 (1-13)	73 (78.5%) 20 (21.5%) 12 (1-21)
LTP	5 (7.8%)	7 (7.5%)
MM>5mm MM<5mm Adenocarcinoma	3 (60%) 2 (40%) 0	6 (85.7%) 1 (14,3%) 4 (57.1%)
Adenocarcinoma in situ	2 (40%)	2 (28.6%)
Squamous cell Carcinoma	0	1 (14.3%)
Lung metastases	3 (60%)	0
Tumor size (mm)	11.8 (5.5–28.9)	12.4 (5.0-37.0)

MM, minimal ablative margin; LTP, local tumor progression.

### 3.2 Complications and length of hospitalization

There were no surgery-related deaths within 30 days after MWA in either group. The incidence of adverse events was higher in the observation group (57.8%, 37/64) compared to the control group (35.5%, 33/93), with a statistically significant difference (p=0.005). The grading of adverse events in the observation group is shown in **Table 3**, and adverse events in both groups are shown in **Table 4**. The incidence of pleural effusion was not statistically significant between the two groups (p=0.76), but the incidence of pneumothorax, hemorrhage, and closed chest drainage was statistically significant. In the observation group, one patient had a postoperative pulmonary embolism and was treated with anticoagulation, and another patient had postoperative gastric pain and acidity and was treated with omeprazole. In the control group, one patient developed bronchospasm, which was relieved with diprophylline; one patient had an acute asthma attack, which was relieved with methylprednisolone combined with dexamethasone; one patient developed circulatory disturbances in the lower extremities, which was improved with mazolotene; and one patient developed a pathologic murmur on auscultation, which was treated with Bucinperazine hydrochloride injection. All patients were discharged safely, with a median length of hospitalization of 5 days for the observation group and 6 days for the control group, with no statistical difference between the two groups (p=0.097).

**Table 3:** Postoperative adverse events after MWA in the observation group.

Complication	Grade	No. (%)
Major		
Pneumothorax	C	21 (32.8%)
Pulmonary embolism	C	1 (1.6%)
Gastric pain and acidity	C	1 (1.6%)
Minor		
Pneumothorax	A	4 (6.3%)
	B	9 (14.1%)
Hemorrhage	A	8 (12.5%)
Pleural effusion	A	4 (6.3%)

**Table 4:** Common complications in two groups.

Adverse events	Observation group	Control group	*p*-value
Overall rates	37 (57.8%)	33 (35.5%)	0.005
Pneumothorax	34 (53.1%)	23 (24.7%)	<0.001
Closed Chest Drainage	21 (32.8%)	11 (11.8%)	0.003
pleural effusion	4 (6.3%)	4 (4.3%)	0.76
Hemoptysis	8 (12.5%)	2 (2.2%)	0.022

### 3.3 Survival rate

**Figure 2:**
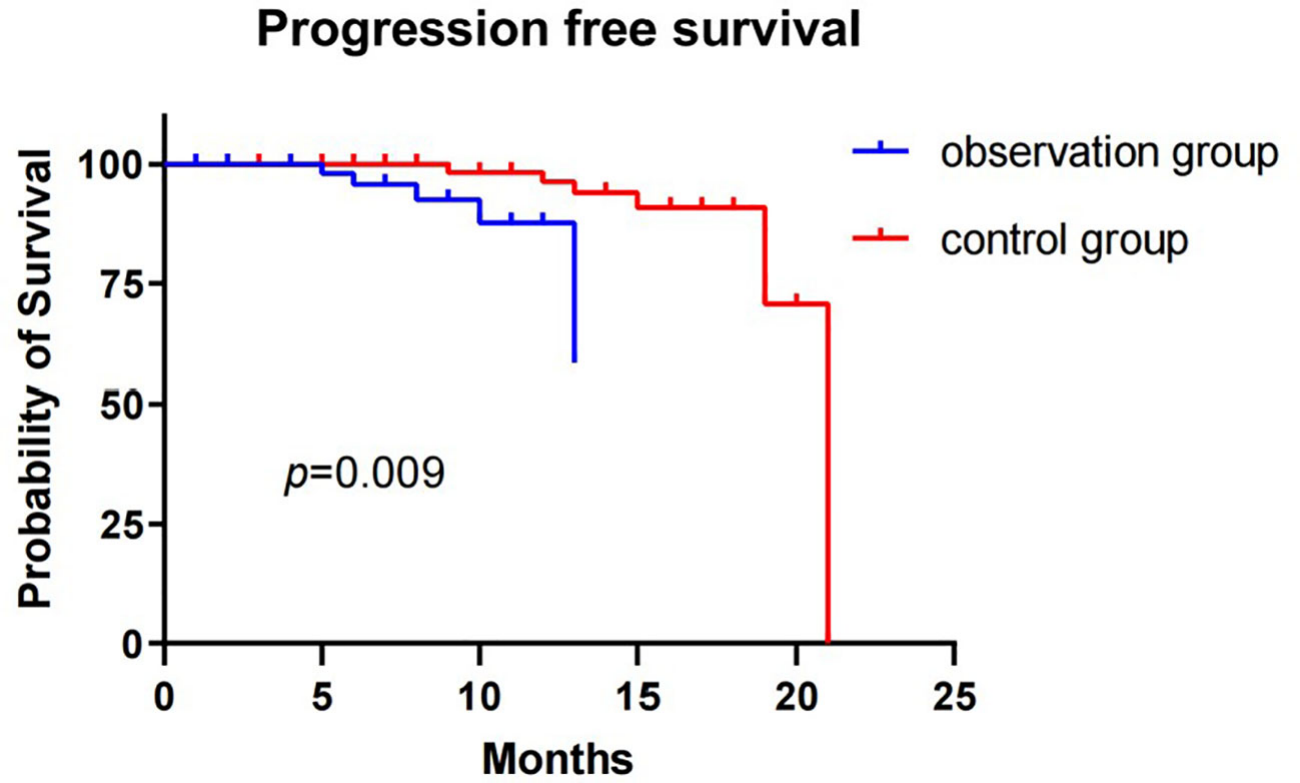
Progression-free survival for observation group and control group patients.

**Figure 3:**
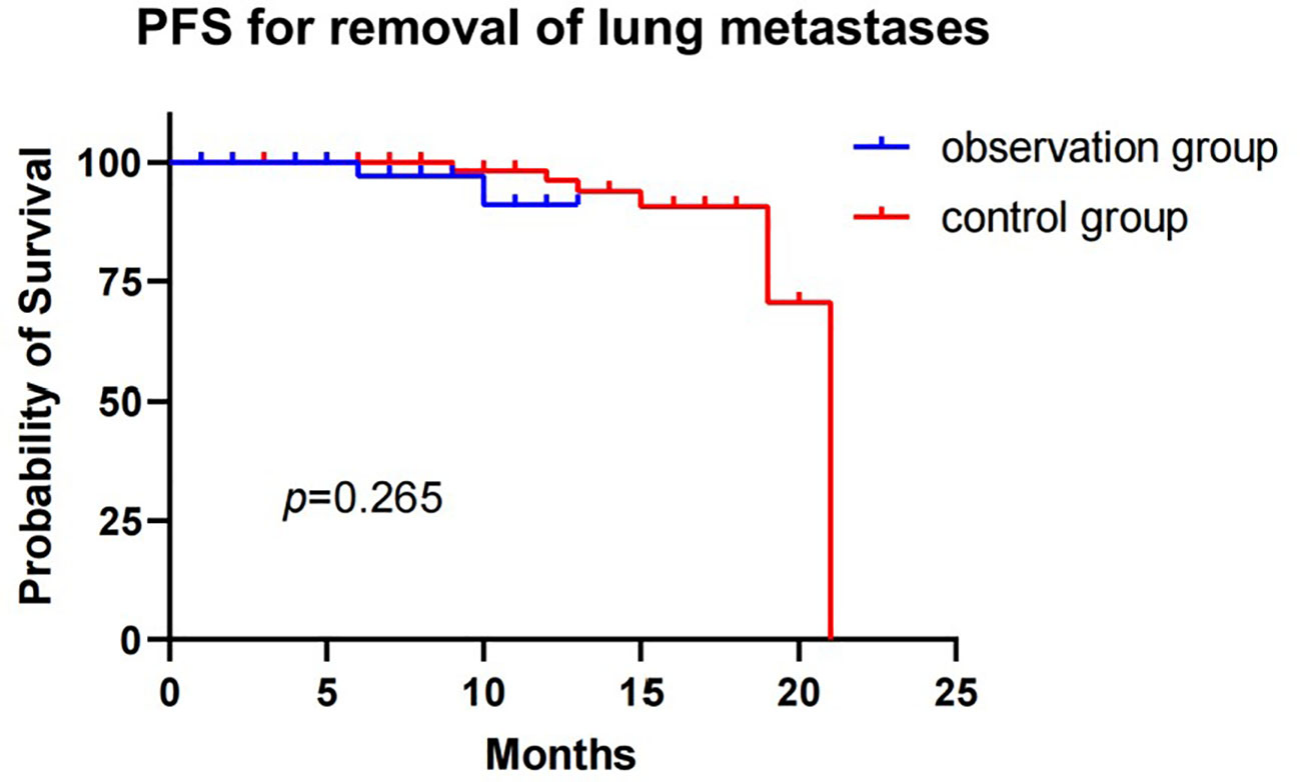
Progression-free survival for observation group and control group patients with removal of lung metastases.

The median PFS of the observation group was not reached, while the median PFS of the control group was 21 months, with a statistically significant difference (p=0.009). No patients were lost during the follow-up period. The 1-year PFS rates were 87.6% and 96.3% in the observation and control groups, respectively (**Figure 2**). When lung metastases were removed from both groups (**Figure 3**), the 1-year survival rates in the observation and control groups were 91.1% and 96.2%, respectively. However, there was no statistically significant difference in PFS between the two groups (p=0.265). Cox regression analysis, which included age, gender, smoking history, and tumor size, showed no statistical significance in both univariate and multivariate analyses (**Table 5**).

**Table 5:** Cox regression analysis of prognostic factors for PFS.

Variables	Univariate analysis		Multivariate analysis	
	HR (95% CI)	p-value	HR (95% CI)	p-value
Age	1.06 (0.97-1.15)	0.223	1.04 (0.95-1.14)	0.428
Gender	1.06 (0.18-6.34)	0.952	0.36 (0.03-5.18)	0.451
Smoking history	0.38 (0.07-2.33)	0.301	0.21 (0.01-3.17)	0.260
Tumor size (mm)	1.03 (0.89-1.20)	0.659	0.98 (0.81-1.18)	0.794

CI, confidence interval; HR, hazard ratio.

## 4 Discussion

This retrospective study evaluated the safety and efficacy of MWA for the treatment of high-risk lung nodules in the observation group by comparing it to patients in the control group. All radical ablations were technically successful, and there were no deaths during tumor ablation or within 30 days of ablation. The LTP rate in this study was 7.8%, which is lower than in previous lung MWA studies such as those by Kurilova et al. (11) (10%) and Zheng et al. (12) (19.1%). This result suggests that MWA may provide reliable efficacy in patients with high-risk lung nodules, including those infected with the Omicron variant.

The treatment of lung malignancies in combination with severe pneumonia remains challenging. Currently, patients with severe pneumonia are typically treated with anti-inflammatory therapy before surgery, and there is a consensus (13) that lung surgery is not recommended within three months of SARS-CoV-2 infection. Previous researches (14, 15) report that patients with lung cancer are more susceptible to COVID-19. While surgical resection is still the standard of care, several studies have shown the effectiveness of ablation in treating high-risk lung nodules and the favorable prognosis of patients (16–18), especially those who are inoperable or refuse surgery due to high-risk conditions. Compared to surgical resection, MWA is minimally invasive, more easily tolerated by patients, better preserves lung function, and has less hospitalization time and cost, making it theoretically more suitable for cases with underlying lung disease and other comorbidities. However, due to the retrospective nature of the study, patients did not routinely undergo pulmonary function testing after ablation, so changes in lung function could not be quantified. Nevertheless, current findings (19, 20) suggest that thermal ablation is relatively safe and does not worsen respiratory insufficiency.

Previous research (21–23) has shown that patients infected with SARS-CoV-2 during the perioperative period after pulmonary resection are at increased risk of postoperative complications and death. Lal et al. (24) found that patients with preoperative SARS-CoV-2 infection had a higher risk of postoperative mortality and pulmonary and ischemic complications. Percutaneous image-guided MWA is a minimally invasive procedure compared to surgery and is therefore theoretically more suitable for cases with underlying lung disease and other comorbidities. However, there are relatively few studies on the safety and efficacy of MWA in treating patients with high-risk lung nodules who have been infected with the Omicron variant. In this study, most of the postoperative complications of MWA were mild, with higher rates of pneumothorax, drainage, and bleeding in the infected group. Among the complications observed, pneumothorax was the most common, with a high incidence of 53.1%, which was higher than the results of other studies (16, 25–30). Most pneumothorax symptoms were mild, and some patients did not require treatment. However, 21/34 (61.8%) patients required drainage, which was also higher than other studies (26, 31, 32). This may suggest that Omicron infection increases the incidence of postoperative complications after MWA. Bleeding was mostly from the needle tract, and 3 patients had minor hemoptysis postoperatively, which was not treated specifically. Despite the relatively high complication rate, none of the patients had serious complications that affected their prognosis, and all patients were discharged safely with or without management. The majority of patients were discharged after 5 days of hospitalization. This suggests that patients infected with Omicron are well-tolerated to receive MWA treatment.

Statistical analysis showed that the 1-year survival rate was 87.6% in the observation group and 96.3% in the control group, suggesting that Omicron infection had an impact on overall survival. However, despite the slightly lower survival rate in the observation group, MWA for high-risk lung nodules in patients with Omicron infection was still effective. The slightly lower survival rate in the observation group could be due to a number of reasons, including potential effects of the virus on the immune system, lung function, or response to treatment (33, 34). However, the differences between the two groups were relatively small and may not be clinically significant. Additionally, the difference in PFS between the two groups was not statistically significant after excluding patients with lung metastases (p=0.265), suggesting that Omicron infection has a relatively small impact on disease progression, especially in patients without lung metastases.

There are several limitations of this study. Firstly, it was a single-center retrospective study with a short median follow-up time, and the limited sample size as well as the great heterogeneity of tumor characteristics may have led to selection bias. Secondly, comparisons with other techniques could not be made due to the limitations of Omicron on other treatments. Finally, lung function tests were not routinely performed after MWA, so we were unable to compare lung function before and after the procedure. Therefore, further prospective, randomized, and multicenter studies with large numbers of patients are needed in the future to obtain more convincing evidence.

## 5 Conclusion

This study demonstrated that preoperative infection with the SARS-CoV-2 Omicron variant in patients undergoing MWA increased the risk of postoperative complications, but did not affect the clinical prognosis of the patients. This suggests that MWA may be a well-tolerated and effective alternative treatment option. We expect that with further research, MWA will become an effective treatment option for more patients with respiratory diseases, especially those with co-infection with Omicron.

## Data Availability

The original contributions presented in the study are included in the article/supplementary material. Further inquiries can be directed to the corresponding author.
